# Physical Properties of Foamed Concrete Based on Plaster Mortar with Polystyrene Granulate and Synthetic Foaming Agent

**DOI:** 10.3390/ma18092115

**Published:** 2025-05-05

**Authors:** Monika Gwóźdź-Lasoń, Wacław Brachaczek, Marta Kadela, Alfred Kukiełka

**Affiliations:** 1 Faculty of Materials, Civil and Environmental Engineering, University of Bielsko-Biala, Willowa 2, 43-309 Bielsko-Biala, Poland; mgwozdz@ubb.edu.pl; 2 Building Research Institute (ITB), Filtrowa 1, 00-611 Warsaw, Poland; a.kukielka@itb.pl

**Keywords:** foamed concrete, foamed mortar, lightweight cellular concrete, pore material, synthetic foaming agent, prepared foam, air void structure, compressive strength, thermal conductivity, multiple regression

## Abstract

According to EU directives, it is necessary to improve the energy consumption of buildings. Therefore, the aim of this study was to improve the physical properties of foamed concrete produced using plaster mortar. For this purpose, polystyrene granulate with a bulk density of 13 kg/m^3^ in amounts of 4, 7, and 10 g per 1 kg of plaster mortar and a foaming agent in amounts of 2, 4, and 6% of the cement mass were used. The density, thermal conductivity coefficient, compressive and flexural strengths, and water absorption coefficient due to capillary action were determined. Based on the obtained results, it can be concluded that the density, thermal conductivity coefficient, and water absorption coefficient due to capillary action decreased with an increase in the content of polystyrene granulate addition, which is a beneficial outcome. However, at the same time, a reduction in mechanical properties was demonstrated. With an increase in the content of the foaming agent, the density and thermal and mechanical properties decreased. The water absorption coefficient due to capillary action increased with the foaming agent content for samples with the addition of polystyrene granulate. However, the coefficient for all the tested samples was lower than that for the base sample.

## 1. Introduction

In accordance with the EU directives, there is a need to improve the energy efficiency of buildings. Appropriate technologies and materials are used to improve energy efficiency in construction [1,2]. One method is to reduce heat transfer through horizontal and vertical partitions. The heat transfer coefficients are lower for porous materials than for materials with a continuous structure. This is because of the enclosure of small portions of gas (air and carbon dioxide) in their volume. However, if the spaces containing gas are too large, convection will begin to occur, which will increase the heat transfer coefficient of a given building material.

This is the basis for the use of foamed concrete in construction [3,4]. Foamed concrete (FC) is classified as lightweight concrete with a minimum air pore volume of 20% in the cementitious mix [5,6,7]. Foamed concrete is produced by adding foam generated mechanically from a foaming agent to a slurry, cement mortar, or concrete mix [8,9]. Foaming agents may be of a protein or synthetic origin [7,10,11]. Other components of foamed concrete include a binder, most often CEM I cement, water, and possibly aggregate [8,12,13]. Usually, foamed concrete does not contain coarse aggregate; therefore it should be called foamed mortar, but this name is not used in practice. Additions (e.g., fly ash, pulverized fuel ash, and granulated blast furnace slag) can also be used as components of foamed concrete [14,15]. However, admixtures are rarely used. The foamed concrete mix is designed according to the assumed density [16]. With the assumed water–cement ratio (w/c), the proportions of sand to cement (s/c), fly ash to cement (a/c), cement, sand, and water content in the foamed concrete mix are determined. Moreover, the volume of foam that must be added to obtain foamed concrete with an assumed density is determined (applies to foamed concrete without admixtures). This is presented in detail in [12,16]. Depending on the composition of the foamed concrete, a wide range of properties can be obtained; hence, they can be widely applied [13,17]. Foamed concrete is primarily used for filling (e.g., voids), relieving, and insulating purposes. Moreover, foamed concrete is used in the construction of running tracks, in road pavement structures [18,19,20], bridge structures [21], and railway track beds [22,23]. Foamed concrete is also used in industrial and residential construction [24,25,26], mainly as a levelling layer, for example under concrete floors [27].

Owing to the air void content of more than 20% in the cement matrix [5,7], foamed concrete is characterized by good thermal properties [28,29,30,31]. The thermal conductivity coefficient ranges from about 0.1 W/(m∙K) to 0.7 W/(m∙K) for foamed concrete with a density ranging from 600 kg/m^3^ to 1600 kg/m^3^ [31,32,33,34,35,36]. For at selected composition of foamed concrete, a thermal conductivity coefficient of up to 0.048 W/(m∙K) can be achieved at a density below 500 kg/m^3^ [11,26]. Gołaszewski et al. [11] determined that the thermal conductivity coefficient for foamed concrete based on Portland cement (OPC) with the addition of metakaolin ranged from 0.058 to 0.076 W/(m·K) with densities ranging from 240 to 380 kg/m^3^. In contrast, for foamed concrete with OPC with the addition of siliceous fly ash (SFA) and calcareous fly ash (CFA), it was 0.057–0.08 W/(m·K) for the same range of densities. Silva et al. [26] obtained a thermal conductivity coefficient of about 0.02 W/(m·K) for foamed concrete with the use of an aerogel addition. While Proshin et al. [37] obtained a thermal conductivity coefficient in the range from 0.06 W/(m∙K) to 0.16 W/(m∙K) for foamed concrete with a density of 200 kg/m^3^ to 650 kg/m^3^. They used polystyrene granulate addition as a component of the foamed concrete.

Because information on foamed concrete with the addition of polystyrene granulate is limited, this study aims to assess the mechanical and thermal properties of hardened foamed mortar based on plaster mortar with polystyrene granulate and a synthetic foaming agent. In addition, the water absorption coefficient due to capillary action was assessed, which has not been investigated thus far. This is particularly important because of the potential applications of foamed concrete as external wall insulation, floor insulation on the ground, and filling in places where water or high humidity may occur. Moreover, in contrast to previous studies of the authors [20,38], foamed concrete was produced based on ready-made plaster mortar used for wall facades. This mixture is novel compared with the basic foamed concrete mixture. In addition, the properties of foamed concrete based plaster mortar have not been published thus far. For the sake of linguistic correctness, this study refers to foamed mortar.

## 2. Materials and Methods

### 2.1. Specimen Preparation

The materials used in this study were plaster mortar, tap water, and foaming agent. A commercial plaster mortar with a density of 945 kg/m^3^ and compressive strength of 4.72 MPa (SEMPRE Farby Sp. z o.o., Bielsko-Biała, Poland) was used. The composition of the plaster mortar is presented in Table 1.

In this study, a commercial liquid polymer admixture with a specific gravity of 1.02 g/cm^3^ was used as the synthetic foaming agent. The synthetic foaming agent (MEEX, Chrzanów, Poland) content was 2.0, 4.0, and 6.0% of the cement mass.

**Figure 1:**
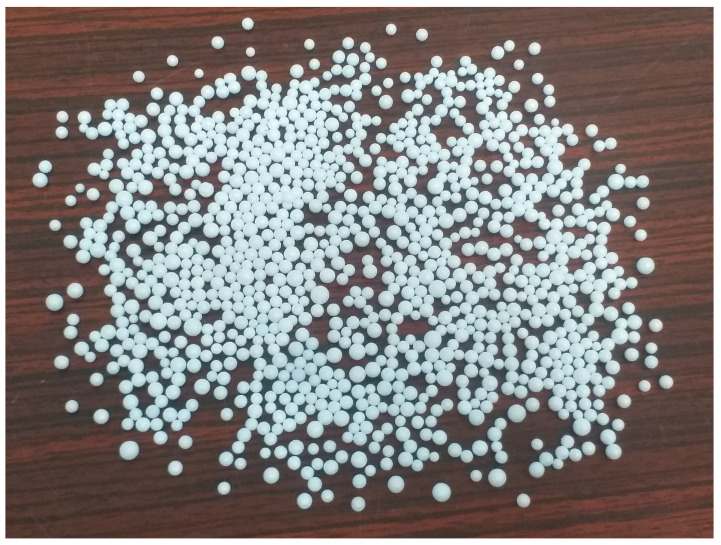
Polystyrene granulate addition used in this study (own photo).

Moreover, an addition of polystyrene granulate (PG) with a bulk density of 13 kg/m^3^ was used (Figure 1).

### 2.2. Mix Composition

Foamed mortar specimens with three different amounts of polystyrene granulate were produced (see Table 2). Based on previous experience, the addition of polystyrene granulate was 10, 7, and 4 g per 1 kg of dry ingredients of plaster mortar. These samples were designated Series 13-1, Series 13-2, and Series 13-3, respectively. The amount of polystyrene granulates added was selected empirically because of the uniform distribution of the polystyrene granulates. The uniform distribution of polystyrene granulates was achieved owing to the appropriate viscosity of the mortar, which prevented the migration of polystyrene granulates towards the upper surface of the sample. This effect was achieved, among others, by using a cellulose thickener as a component of the plaster mortar (Table 1) and determining the appropriate amount of mixing water and mortar consistency. This was empirically determined in cooperation with the mortar manufacturer.

The polystyrene granulate content was selected so that the volume of dry ingredients in 1 kg of plaster mortar and the volume of granulate were the same. A volume ratio of 1:1 was obtained for polystyrene granulate in the amount of 7 g. In other cases, the amount of added PG was proportionally reduced or increased.

### 2.3. Mix Production

**Figure 2:**
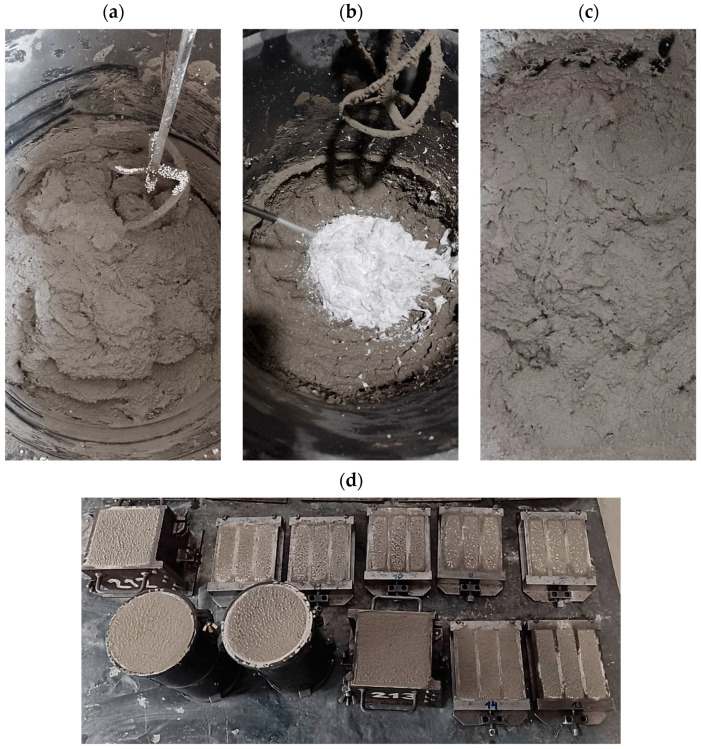
Sample production process: (**a**) mixing plaster mortar with polystyrene granulate addition, (**b**) adding foam to plaster mortar with polystyrene granulate addition, (**c**) foamed mortar mix, (**d**) selected samples with foamed mortar in steel molds (own photos).

The foamed mortar mix was produced according to the preforming method. First, plaster mortar and water were mixed. After six minutes, the polystyrene granulate was added to the mix, and all the components were mixed for one minute (Figure 2a). Next, the stable foam was added to the mix, and all the components were mixed (Figure 2b,c). The foam was created using a foam generator (Techkar S.K.A., Brzesko, Poland). The ready-mixed foamed mortar was cast in steel molds (Figure 2d) to produce specimens of the appropriate dimensions and required numbers for the individual tests in this study; the exact number and dimensions of the specimens are given in the description of each test (see Section 2.4).

After casting, all specimens were covered with cellophane to protect against water evaporation and ensure the best bonding conditions [40]. The samples were stored in a curing room at 20 ±1 °C. After 24 h, the samples were removed from the steel molds and stored for another 14 days in a curing room at 20 ±1 °C and 95% humidity. Subsequently, the samples were stored under ambient conditions of 20 ±1 °C and 60 ±10% humidity. Sample testing was performed after 28 days of curing. Two cases were considered: in the first, a mortar with the addition of polystyrene granulate, and in the second, a foamed mortar with the addition of polystyrene granulate. The results were compared with those of the base sample (mortar without the addition of polystyrene granulate or foaming agent).

### 2.4. Methodology

#### 2.4.1. Density

The density of the foamed mortar specimens was measured with 40 × 40 × 160 mm standard beams, as per PN-EN 1015-10 [41]. Three naturally dried samples were examined for each mix.

#### 2.4.2. Thermal Conductivity

**Figure 3:**
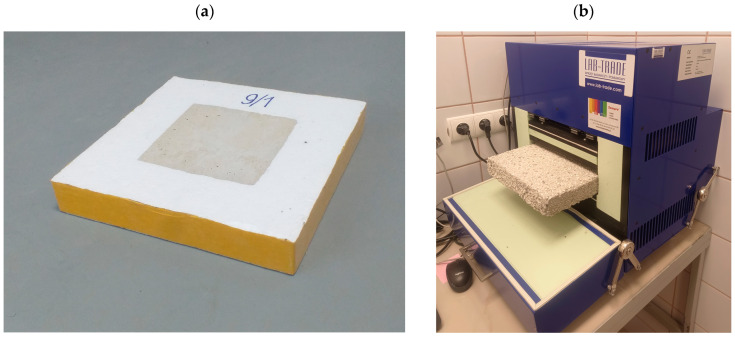
Test of thermal conductivity coefficient: (**a**) prepared foamed concrete sample, (**b**) testing apparatus (own photos).

The thermal conductivity was measured on samples 150 × 150 × 50 mm (Figure 3). The device for testing thermal conductivity is based on the relative method of measuring the thermal conductivity coefficient, which consists of measuring the ratio of the thermal resistance of the tested samples to that of the reference samples. The reference material was a resin-bonded glass fiber board with dimensions of 300 × 300 × 34.2 mm and a density of 76 kg/m^3^. The thermal conductivity coefficient of the reference sample was equal 0.03048 W/(m·K).

The measurements were performed at a constant average sample temperature and constant temperature difference on the upper and lower surfaces of the sample, and at a constant and uniform heat flux passing through the sample measurement area. Three samples were tested per mix. Prior to testing, samples were dried to a constant mass.

#### 2.4.3. Flexural Strength

**Figure 4:**
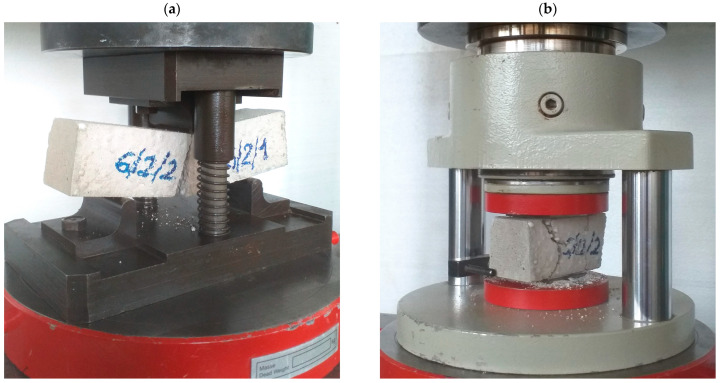
Test of the mechanical properties of foamed mortar: (**a**) flexural strength, (**b**) compressive strength (own photos).

The flexural strength was determined using the three-point bending test of beams with dimensions of 40 × 40 × 160 mm (Figure 4a) in accordance with PN-EN 1015-11 [42]. Three naturally dried samples were tested for each mix.

#### 2.4.4. Compressive Strength

The compressive strength was determined according to PN-EN 1015-11 [42]. The beam halves obtained after the flexural strength tests were used in the tests (Figure 4b).

#### 2.4.5. Water Absorption Coefficient Due to Capillary Action

**Figure 5:**
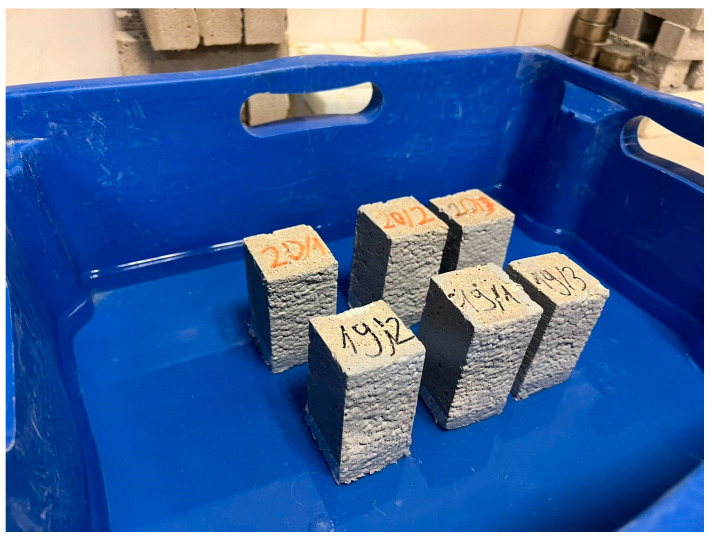
Test of the water absorption coefficient due to capillary action of foamed mortar (own photo).

The water absorption coefficient due to capillary action was measured on samples of 40 × 40 × 160 mm (Figure 5) for ordinary (non-renovation) mortars according to PN-EN 1015-18 [43]. The water absorption coefficient due to capillary action was determined according to Formula (1).

*C* = 0.1∙(*M*_2_ − *M*_1_) [kg/(m^2^·min^0.5^)], (1)

where

*M*_1_ is the sample weight after storage in water for 10 min [g] and
*M*_2_ is the sample weight after storage in water for 90 min [g].

## 3. Results and Discussion

**Figure 6:**
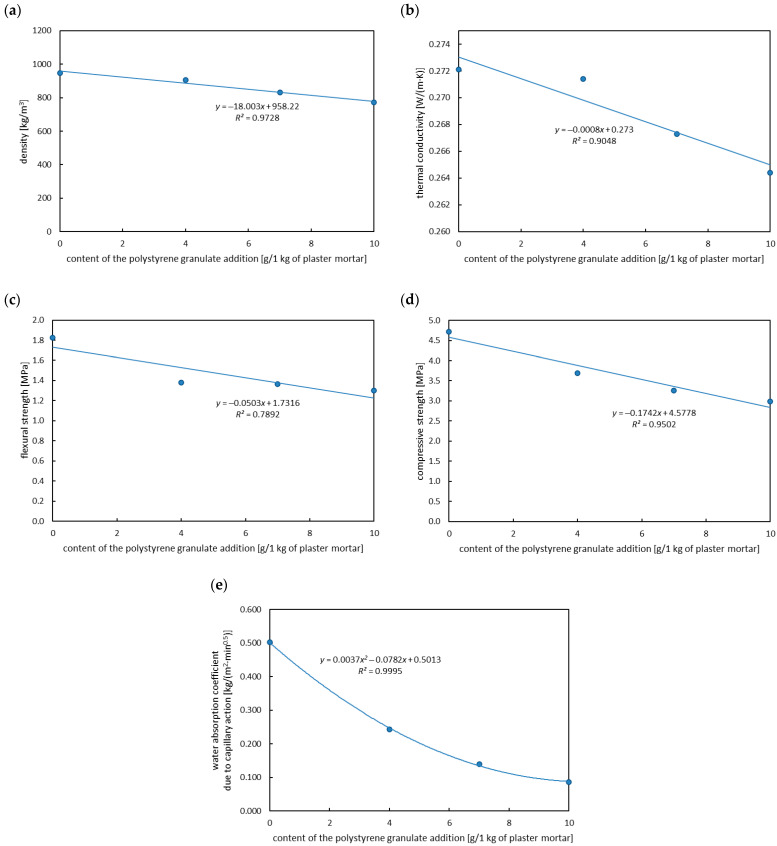
Effect of polystyrene granulate addition on the physical properties of plaster mortar: (**a**) density, (**b**) thermal conductivity, (**c**) flexural strength, (**d**) compressive strength, (**e**) water absorption coefficient due to capillary action.

In the first case, the effect of the addition of polystyrene granulate on the plaster mortar properties (without a foaming agent) was analyzed. The density (Figure 6a), thermal conductivity coefficient (Figure 6b), and water absorption coefficient due to capillary action (Figure 6e) decreased with an increase in the content of polystyrene granulate addition, which is a beneficial outcome. However, at the same time, a reduction in mechanical properties was demonstrated (Figure 6c,d). A significant decrease in the water absorption coefficient due to capillary action and mechanical properties (flexural and compressive strength) compared to the base sample was obtained (see Table 3).

**Figure 7:**
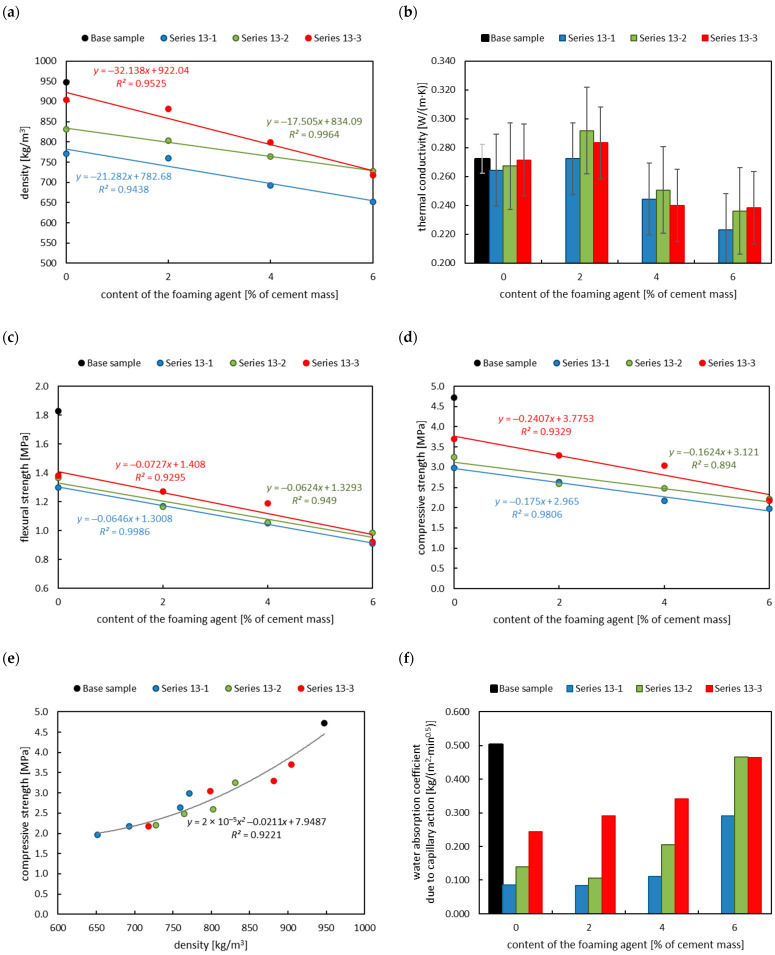
Effect of polystyrene granulate addition on the physical properties of foamed mortar: (**a**) density, (**b**) thermal conductivity, (**c**) flexural strength, (**d**) compressive strength, (**e**) compressive strength depending on density, (**f**) water absorption coefficient due to capillary action.

In the second case, the effect of the foaming agent content on the properties of foamed mortar with different contents of polystyrene granulate addition was analyzed. It can be seen that the effect of the foaming agent content on the analyzed physical properties of the foamed mortar with the addition of polystyrene granulate was ambiguous (Figure 7).

Figure 7a presents the results for the density of the hardened foamed mortar with the addition of polystyrene granulate for a foaming agent contents of 2.0, 4.0, and 6.0% of cement mass. It can be observed that the density of foamed mortar decreased with increasing foaming agent content, and this correlation is linear. The foam volume commonly created air voids and resulted in a lower density [17]. These results were consistent with the observations for foamed concrete with a basic composition [6,7,20,38]. The lowest thermal conductivity coefficient of the foamed mortar was obtained for the highest foaming agent content, regardless of the content of polystyrene granulate. The thermal conductivity coefficient obtained for the foaming agent content of 2% of the cement mass was higher than that of the base sample of the plaster mortar and the base samples of the plaster mortar with the addition of polystyrene granulate (see Figure 7b and compare with Figure 6b). Owing to the high standard deviation of the base samples, it can be stated that the obtained thermal conductivity coefficient for the samples of foamed concrete with polystyrene granulate addition and foaming agent at the amount of 2% was equal to or lower than that of the base samples. The thermal conductivity coefficient obtained was higher than that obtained for the same density of foamed concrete by Proshin et al. [37]. In the present study, for the density of 650 kg/m^3^, the thermal conductivity coefficient was 0.223 W/(m∙K), whereas Proshin et al. [36] obtained 0.16 W/(m∙K). This is probably related to the composition of the foamed concrete mix and indicates the need for further research.

The mechanical properties of foamed concrete are directly related to its density and foaming agent content [13,20,38]. The higher the foaming agent content (lower density), the lower the flexural and compressive strength [3,17]. The flexural (Figure 7c) and compressive strengths (Figure 7d) of the foamed mortar were halved with a foaming agent content of 6% of the cement mass compared to the base mortar. Figure 7e presents the correlation between density and compressive strength for foamed mortars with different foaming agent contents and polystyrene granulate addition. It can be seen that this correlation is exponential. This is consistent with our previous results [20,38] and the results of other researchers [17,40,44,45,46,47] for other compositions of foamed concrete mixes. The correlation equation obtained in this study was found to be in good agreement (for all results) with the results of previous studies on foamed concrete based on basic composition [20,38]. Moreover, an interesting observation is that the water absorption coefficient due to capillary action is lower for foamed mortar produced with a higher content of foaming agent, regardless of the content of polystyrene granulate addition (see Figure 7f). The lowest water absorption coefficients due to capillary action were obtained for foamed concrete produced with the addition of polystyrene granulate with the highest analyzed content.

To determine the quantitative relationship between the variables for the amount of the addition of polystyrene granulate (X1) and foaming agent (X2), and the compressive strength, flexural strength, thermal conductivity coefficient, density, and water absorption coefficient due to capillary action of hardened foamed concrete, multiple regression was used. A linear model was used according to Formula (2).

Y = *b*_0_ + *b*_1_∙X_1_ + *b*_2_∙X_2_ + ε, (2)

where

*b*_i_ is the model parameter (regression coefficient) describing the impact of the *i*-th variable and
ε is the random component (standard error of estimation *Se*).

Verification of the models was performed by assessing the significance of the regression function, and regression coefficients, and based on the analysis of residuals. The significance of the models was tested using an *F*-test at a probability level of *p* = 0.05. The multiple correlation coefficient *R* and *R^2^* index were adopted as measures of interdependence between one of the variables and the other variables treated together. Table 4 presents the results of the multiple regression.

In each case, the *F*-values were greater than the critical values of the *F*–Snedecor distribution at a significance level of 0.05 (*F(2,9)_cr_* = 4.26). The significance of the models was also confirmed by the *p*-values, which were less than the generally accepted value of 0.05. If the correlation coefficient is *R* ≈ 1, there is a strong linear relationship between the variables. Next, the significance of the regression coefficients was assessed, see Table 5.

It can be observed that in the case of testing the variation of density, compressive and flexural strength and water absorption coefficient due to capillary action, the *p*-values for partial regression coefficients were significant (*p* < 0.05).

In the case of testing the variation in the thermal conductivity coefficient *λ*, the independent variable of the content of the addition of polystyrene granulate (X1) was insignificant. The independent variable of the foaming agent content (X2) was highly significant. However, that the variation in the dependent variable was explained by 58% (*R_corrected_* = 0.58724843). Such a model may be acceptable in complex systems in which other factors may have an influence. For the λ coefficient, a well-fitted model was obtained in the form of Formula (3):

*λ* = 0.282919 − 0.008423·X2 ± 0.01544. (3)

**Figure 8:**
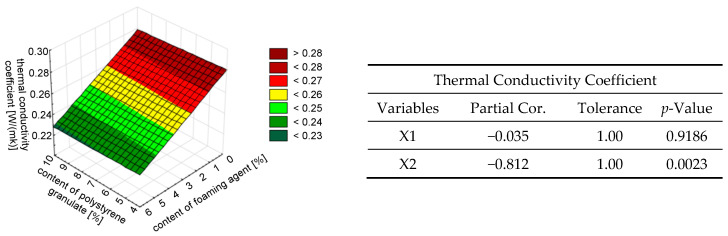
The effect of the simultaneous addition of polystyrene granulate X1 [%] and foaming agent X2 [%] on the thermal conductivity coefficient of foamed concrete *λ* described by a linear model.

**Figure 9:**
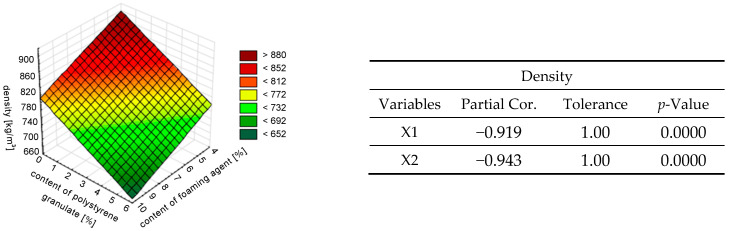
The effect of the simultaneous addition of polystyrene granulate X1 [%] and foaming agent X2 [%] on the density of foamed concrete *γ* described by a linear model.

**Figure 10:**
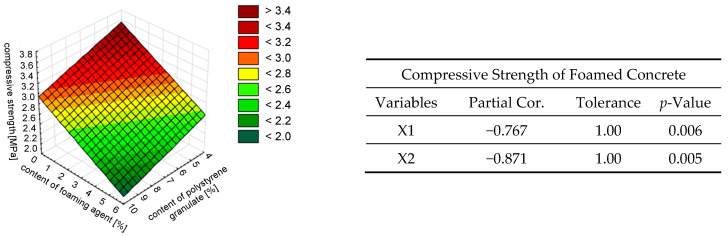
The effect of the simultaneous addition of polystyrene granulate X1 [%] and foaming agent X2 [%] on the compressive strength of foamed concrete *f_c_*, described by a linear model.

**Figure 11:**
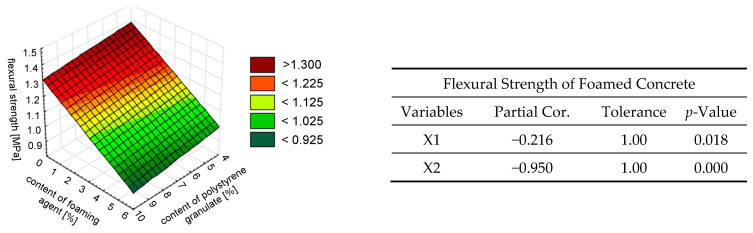
The effect of the simultaneous addition of polystyrene granulate X1 [%] and foaming agent X2 [%] on the flexural strength of foamed concrete *fc_,fl_*, described by a linear model.

**Figure 12:**
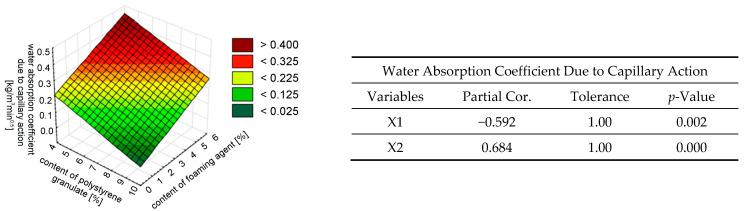
The effect of the simultaneous addition of polystyrene granulate X1 [%] and foaming agent X2 [%] on the water absorption coefficient due to capillary action *C*, described by a linear model.

In the next step, redundancy (lack of collinearity) between the independent variables was determined. The *R*^2^ coefficient, tolerance, and partial and semi-partial correlations were calculated for each variable. Semi-partial correlation (also called partial correlation) describes the relationship between two variables while controlling for the impact of one or more additional variables. To illustrate the trends and graphically represent the structure of the data, surface plots were constructed (see Figure 8, Figure 9, Figure 10, Figure 11 and Figure 12). Figure 8 presents the variation of the thermal conductivity coefficient *λ* and the results of the redundancy test. Analyzing the results, it was found that the semi-molecular correlation for the variable of the addition of polystyrene granulate (X1) was very small, amounting to −0.02. This indicates a weak correlation between this variable and the dependent variable *λ*. However, the variable of the content of the foaming agent (X2) is highly correlated and explains 82% of the variation in the *λ* coefficient.

A high semi-molecular correlation of both independent variables (X1 and X2) was observed for all other analyzed properties of foamed concrete (i.e., density, compressive and flexural strength, and water absorption coefficient due to capillary action).

The trends in the density variation of foamed concrete are presented in Figure 9. The semi-partial correlations between the independent variables and density were comparable and amounted to −0.943 for the foaming agent content (X2) and −0.919 for the polystyrene granulate addition content (X1). This means that the variability in the contents of these components affected the density variation of foamed mortar to a comparable extent (the strengths of the interaction of both variables were comparable, but the variables were inversely correlated).

Figure 10 presents a surface graph of the variation in the compressive strength of hardened foamed concrete. During the redundancy test, it was found that the semi-partial correlations between the independent variables and density were diverse, amounting to −0.871 for the foaming agent content (X2) and −0.767 for the content of the polystyrene granulate addition (X1). This means that the effect of the variation in the amount of foaming agent on the variation in the compressive strength of foamed concrete was greater (the strength of the effect of this variable was greater than that of the content of the polystyrene granulate addition). However, these variables were found to be inversely correlated.

In the study of flexural strength variation, a strong correlation with the variable of the foaming agent content (X2) was demonstrated, with a level of 95%. The impact of the content of the polystyrene granulate addition was insignificant (see Figure 11). The trends of the simultaneous influence of the foaming agent content (X2) and the addition of polystyrene granulate (X1) on the water absorption coefficient due to capillary action are presented in Figure 12.

By analyzing the partial correlations (Figure 12), it can be concluded that both variables were similarly correlated. This indicates a strong relationship between both independent variables, X1 and X2, and the water absorption coefficient due to capillary action, wherein the variable of the foaming agent content (X2) was inversely correlated.

Thus, the content of the foaming agent had a greater impact on all analyzed properties of the foamed mortar with the addition of polystyrene granulate, as shown in Figure 8, Figure 9, Figure 10, Figure 11 and Figure 12. This was due to the fact that the foaming agent directly influenced the formation of air pores in the cement matrix. The exception was the density. It was estimated that the addition of polystyrene granulate affected the density of the foamed mortar in a manner similar to that of a foaming agent. This can be related to the ‘comparable weight’ of the air and polystyrene granulate, which is light.

The addition of polystyrene granulate addition to the foamed mortar did not significantly affect the thermal conductivity. However, it reduced the density (to the same extent as foaming; see Figure 7a and Figure 9) and improved the water absorption coefficient due to capillary action (Figure 7f and Figure 12) of the foamed mortar compared to the base sample of the plaster mortar.

## 4. Conclusions

The aim of this study was to assess the physical properties of hardened foamed mortars with polystyrene granulate addition. In this study, foamed concrete was produced using plaster mortar. Polystyrene granulate with a bulk density of 13 kg/m^3^ in amounts of 4, 7, and 10 g per 1 kg of plaster mortar and a foaming agent in amounts of 2, 4, and 6% of cement mass were used. The following key conclusions were drawn based on the results of this experimental investigation:

- The density, thermal conductivity coefficient, and water absorption coefficient due to capillary action decreased with an increase in content of polystyrene granulate addition. This is a beneficial outcome, particularly because of its potential applications. However, at the same time, a reduction in mechanical properties was demonstrated.
- The water absorption coefficient due to capillary action of all samples of foamed mortar was lower than that of the base sample of the plaster mortar. However, this coefficient increased with increasing foaming agent content for samples with the addition of polystyrene granulate.
- Based on the results of statistical analyses, it was estimated that the addition of polystyrene granulate affected the density of foamed mortar in a manner similar to that of a foaming agent. Both components can be used to reduce the density compared with the base sample of the plaster mortar.
- The addition of polystyrene granulate affected the density, compressive strength, and the water absorption coefficient due to capillary action of the foamed mortar to the same extent as that of the foaming agent. With increasing content of polystyrene granulate addition, the water absorption coefficient decreased.
- The polystyrene granulate had a smaller effect on the reduction in compressive strength than the foaming agent content.
- The effect of the polystyrene granulate on the thermal conductivity coefficient was insignificant. However, it reduced the density (to the same extent as the foaming agent) and improved the water absorption coefficient due to capillary action of the foamed mortar compared to the base sample of plaster mortar.

Based on the conducted research, it can be concluded that the addition of polystyrene granulate and foaming agents affected the physical properties of foamed concrete.

The use of the polystyrene granulate addition had a beneficial effect on the natural environment owing to the improvement in the thermal insulation of the plaster mortar used at wall elevations. In the future, it is planned to use recycled polystyrene granulate addition, which will have a positive impact on improving environmental protection.

Moreover, it is planned that the observed inverse correlation between polystyrene granulate content and mechanical properties will be elaborated further, perhaps through additional theoretical insights or modelling. For this purpose, additional studies will be carried out, including an examination of the structure of foamed concrete using an SEM microscope.

## Figures and Tables

**Table 1:** The composition of the plaster mortar [39].

Component	Quartz Sand 0.0–0.5 mm	Cement CEM I 42.5R	Filling Aggregate (Limestone Flour)	Lime	Expanded Perlite	Redispersible Polymer Powder (Polyvinyl Acetate Modified with Ethylene)	Thickener (Hydroxyethyl Methyl Cellulose with a Viscosity of Approx. 15,000 mPa∙s)	Foaming Agent Based on Alpha-Olefin Sulfate and Sodium Salt
Unit (vol. %)	63.02	20.00	10.00	5.00	1.20	0.50	0.25	0.03

**Table 2:** Mix proportions of one batch.

Mix Symbol	Plaster Mortar [kg]	Polystyrene Granulate Addition [g]	Water [kg]	Foaming Agent [% of Cement Mass]
Series 13-1	12	120	3.120	0.0
	12	120	3.072	2.0
	12	120	3.024	4.0
	12	120	2.976	6.0
Series 13-2	12	84	3.120	0.0
	12	84	3.072	2.0
	12	84	3.024	4.0
	12	84	2.976	6.0
Series 13-3	12	48	3.120	0.0
	12	48	3.072	2.0
	12	48	3.024	4.0
	12	48	2.976	6.0

**Table 3:** Percentage decrease in physical properties of plaster mortar with polystyrene granulate addition compared to the base plaster mortar.

No.	Decrease [%]				
	Density	Thermal Conductivity Coefficient	Flexural Strength	Compressive Strength	Water Absorption Coefficient Due to Capillary Action
	[kg/m^3^]	[W/(m∙K)]	[MPa]	[MPa]	[kg/(m^2^·min^0.5^)]
Series 13-1	18.6	2.8	28.8	36.8	82.8
Series 13-2	12.2	1.8	25.4	31.1	72.2
Series 13-3	4.5	0.3	24.5	21.7	51.6

**Table 4:** Multiple regression results.

Dependent Variable	*F(2,9)*	*p*	*S_e_*	*R*	*R* ^2^
Thermal conductivity coefficient [W/(m∙K)]	8.8252	<0.0076	0.01544	0.81381	0.58248
Density [kg/m^3^]	60.383	<0.0001	21.565	0.96469	0.91523
Flexural strength [MPa]	85.290	<0.0000	0.04034	0.97461	0.93874
Compressive strength [MPa]	20.601	<0.0004	0.24348	0.90593	0.78088
Water absorption coefficient due to capillary action [kg/(m^2^∙min^0.5^)]	0.9052	<0.0004	0.06505	0.90521	0.77924

**Table 5:** Regression results.

Dependent Variable	*b*_0_	*p*-Value	*b*_1_	*p*-Value	*b*_2_	*p*-Value
Thermal conductivity coefficient [W/(m∙K)]	0.2829	0.000	−0.00041	0.818	−0.0084	0.002
Density [kg/m^3^]	970.875	0.000	−17.7917	0.005	−23.583	0.000
Flexural strength [MPa]	1.44192	0.000	−0.01375	0.017	−0.0663	0.000
Compressive strength [MPa]	3.97241	0.000	−0.10291	0.005	−0.1673	0.000
Water absorption coefficient due to capillary action [kg/(m^2^∙min^0.5^)]	0.33815	0.000	−0.03204	0.002	0.0406	0.000

## Data Availability

The original contributions presented in this study are included in the article. Further inquiries can be directed to the corresponding authors.
